# Responsiveness of Carnosine Homeostasis Genes in the Pancreas and Brain of Streptozotocin-Treated Mice Exposed to Dietary Carnosine

**DOI:** 10.3390/ijms19061713

**Published:** 2018-06-09

**Authors:** Amilcare Barca, Francesca Gatti, Daniela Spagnolo, Stefania Ippati, Carla Vetrugno, Tiziano Verri

**Affiliations:** 1 General Physiology Laboratory, Department of Biological and Environmental Sciences and Technologies, University of Salento, 73100 Lecce, Italy; danielaspagnolo9@gmail.com (D.S.); carlottavetrugno@virgilio.it (C.V.); 2 K.G. Jebsen Inflammation Research Centre, University of Oslo, 0318 Oslo, Norway; francesca.gatti@medisin.uio.no; 3 IRCCS—Istituto di Ricerche Farmacologiche Mario Negri, Department of Neuroscience, 20156 Milan, Italy; stefania.ippati@marionegri.it

**Keywords:** dietary carnosine, di-tripeptides, Pept2/Slc15a2 oligopeptide transporter, Cndp2 dipeptidase, hyperglycemia, diabetes, pancreas, insulin, diabetes mouse models, homeostasis of small peptides

## Abstract

In excitable tissues, the endogenous dipeptide carnosine (CAR, β-Ala-l-His) sustains homeostatic responses to various challenges. By eliciting hypoglycemic effects via actions on the autonomic nervous system and protection of pancreatic beta-cells, CAR is also relevant in diabetes. We investigated the expression of genes involved in CAR biosynthesis, degradation, and membrane transport pathways, in the pancreas and brains of mice treated with streptozotocin (STZ) and then exposed to dietary CAR. We induced hyperglycemia by STZ intraperitoneal injections; then, STZ-treated mice received drinking water with or without CAR for two weeks. We report that CAR administration elicits beneficial effects on blood glucose levels and weight loss in STZ-treated mice and, remarkably, on the insulin gene products in the pancreas, preserving gene expression from STZ challenge. Also, we describe mRNA downregulation of the *Slc15a2*/*Pept2* (dipeptide transporter) and *Cndp2* (intracellular dipeptidase) genes in the pancreas of hyperglycemic mice, and dysregulation of *Carns1* (CAR synthase), *Pept2* and *Cndp2* in brains; interestingly, dietary CAR elicits counteracting effects. These expression patterns associate with variations of CAR content in tissues of mice. Overall, our report suggests a direct role of CAR in the diabetes-affected pancreas and in the diabetes-targeted CNS, proposing (dys)regulation of CAR’s homeostasis as a marker condition.

## 1. Introduction

Carnosine (CAR, β-alanine-l-histidine) and other CAR-derived histidine-containing dipeptides (HCDs) are endogenous dipeptides known for sustaining multifunctional homeostatic responses to various challenges in vertebrates’ excitable tissues, i.e., muscle and CNS, in which CAR and its derivatives are abundant. CAR is acknowledged to act as a pH buffer, divalent ion chelator, ROS-counteracting antioxidant, and inhibitor of advanced glycation end-products [1,2,3]. In virtue of its effects, CAR and other HCDs have been investigated in the context of diabetes complications affecting many organs (e.g., CNS, eye, kidney, liver). Hypoglycemic effects of CAR have been suggested, possibly due to interactions with the autonomic nervous system [4,5] and, also, to the ability in preserving pancreatic beta-cells [3,6,7]. In several mouse models of hyperglycemia, CAR treatments elicit protective effects on blood glucose levels and plasmatic insulin levels; also, hepatic damage, plasmatic fibronectin levels, and dyslipidemia have been found to be reduced [6,8,9]. The diabetes-counteracting effects of CAR have also been investigated to a certain extent in human trials. Oral supplementation with CAR resulted in a significant amelioration of oxidative stress, glycemia control, and renal function in pediatric diabetic patients [10], and CAR is also acknowledged of attenuating fasting glucose, triglycerides, AGEs, and tumor necrosis factor-alpha levels in patients with type 2 diabetes [11]. Nevertheless, CAR’s potential as a dietary supplement in human is still uncertain [12], due to the presence of CAR-degrading activity by dipeptidases (carnosinases), intracellular or circulating in the blood [13,14]. Several studies indicate that CAR prevents diabetes-associated nephropathic complications by counteracting glomerular apoptosis and podocyte loss, and, interestingly, suggest the involvement of the CAR/carnosinase dual system in the diabetes-related onsets. In diabetic mice, the increase of renal CAR-degrading enzymatic activity is described as mediated by reactive metabolites. Remarkably, the *CNDP1* and *CNDP2* human genes, coding for the two major CAR-degrading dipeptidases (serum and intracellular carnosinase, respectively), are positioned in susceptible loci for type I and II diabetes within human chromosomes [3,15,16,17,18,19,20,21]. To date, the biological role of CAR in diabetes has not been sufficiently elucidated. Despite analytical data on CAR levels and carnosinase activity in blood, plasma, or serum being available, the characterization of the expression of genes involved in CAR homeostasis is still missing in the pancreas and is not exhaustive in various diabetes-targeted organs.

Here, in mice with overt hyperglycemia induced by streptozotocin (STZ) and exposed to dietary CAR supplementation, we analyzed the expression of a core gene network regulating CAR homeostasis (i.e., the genes coding for the Carns1 CAR synthase, Cndp1 and Cndp2 extra- and intracellular CAR dipeptidases, and the Slc15a2/Pept2 H^+^/oligopeptide transporter) and the presence of CAR content in the pancreas. In parallel, we analyzed the ability of CAR in exerting beneficial effects on blood glucose levels and weight loss in STZ-treated mice, and in counteracting the STZ-induced impairment of insulin gene expression in the pancreatic tissue. We also performed our analyses in a diabetes target district of the CNS, where CAR is investigated in relation to oxidative stress, ageing and neurodegeneration [3,12,14,22,23,24], identifying the impact of STZ treatments on CAR homeostasis in brains of mice and the counteraction exerted by dietary CAR.

## 2. Results

### 2.1. Model Validation: Body Weight and Glucose Levels in STZ-Treated Hyperglycemic Mice that Underwent CAR Oral Administration for Two Weeks

**Figure 1:**
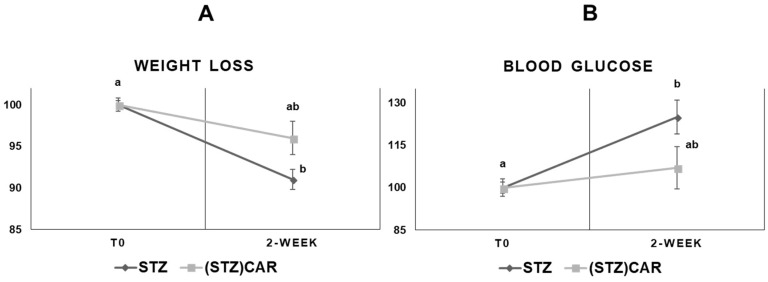
Weight loss (**A**) and blood glucose levels (**B**) in mice that underwent STZ treatments in the absence and presence of CAR oral administration. Relative (%) weight and blood glucose concentrations after two weeks from overt hyperglycemia [T0, 100%]. Overt hyperglycemia refers to the condition of STZ-treated mice showing sustained blood glucose concentration ≥ 250 mg/dL for at least seven days (see Materials and Methods Section 4.1). STZ: STZ-treated, hyperglycemic mice; (STZ)CAR: STZ-treated mice underwent a two-week CAR administration (1 g/L in drinking water). Values refer to the mean (±S.E.M.) of *n* = 5 individuals. Statistical analysis: *t*-Student; different letters indicate significantly different values (*p* < 0.05).

To assess the systemic effects of STZ treatments, blood glucose concentration and body weight were monitored for two weeks starting from overt hyperglycemia (T0) in hyperglycemic mice (STZ) and in STZ-treated mice undergoing CAR oral administration ((STZ)CAR). As shown in Figure 1A, STZ mice exhibited a 9% weight loss consequent to treatment, while (STZ)CAR mice exhibited halved body weight reduction, i.e., 4%. In parallel, glucose levels (Figure 1B) increased up to 125% in STZ mice but were raised no more than 107% in (STZ)CAR mice, as detected two weeks after overt hyperglycemia (T0 = 100%).

### 2.2. Model Validation: Expression of the Insulin Gene Products in the Pancreas of STZ-Treated Hyperglycemic Mice that Underwent CAR Oral Administration for Two Weeks

**Figure 2:**
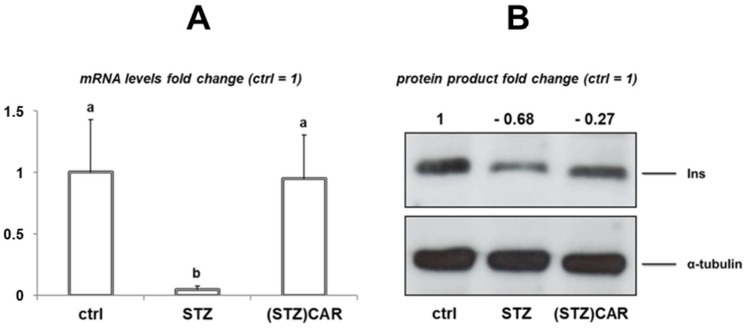
Expression of the insulin gene products in the pancreas tissue. **2A**: qPCR analysis of the Ins1 mRNA. Ctrl: untreated control mice; STZ: hyperglycemic mice; (STZ)CAR: hyperglycemic mice orally administered with CAR. Expression of the mRNA target is obtained from the value of the 2^−Δ*C*t^ function (*C*_t_, Threshold cycle) normalized respect to the 28S RNA according to Schmittgen and Livak [25]. Values refer to the mean data (±S.E.M.) from *n* = 5 individuals and are expressed as fold change with respect to control (fold change 1). Statistical analysis: one-way ANOVA, post hoc test by Bonferroni; different letters (a, b) indicate significantly different values (*p* <0.05). **2B**: representative Western blot assay for the insulin protein product (Ins) in the protein extract from the pancreas. For each sample lane, the densitometric quantification of the Ins product band refers to the mean data from *n* = 3 individuals. Mean values have been normalized respect to the corresponding α-tubulin band.

In STZ and (STZ)CAR mice the expression of the Ins1 mRNA was studied by qPCR, compared to the untreated controls. In the STZ hyperglycemic mice group, a significant downregulation (negative fold change of −0.95 ± 0.03) was detected compared to the control (ctrl; fold change 1 ± 0.43), whereas in the (STZ)CAR mice no significantly different mRNA levels were detected (negative fold change −0.05 ± 0.35; Figure 2A). Accordingly, the expression of the insulin protein product in the pancreatic tissue of the STZ experimental group was strongly down-regulated (−68 ± 0.15 negative fold change) vs. the control (Figure 2B), whilst in the (STZ)CAR group the downregulation occurred to a lesser extent (−0.27 ± 0.12 negative fold change).

### 2.3. Expression Variations of mRNAs Related to CAR Homeostasis in the Pancreas and Brain of STZ-Treated Mice that Underwent (Dietary) CAR Oral Administration for Two Weeks

**Figure 3:**
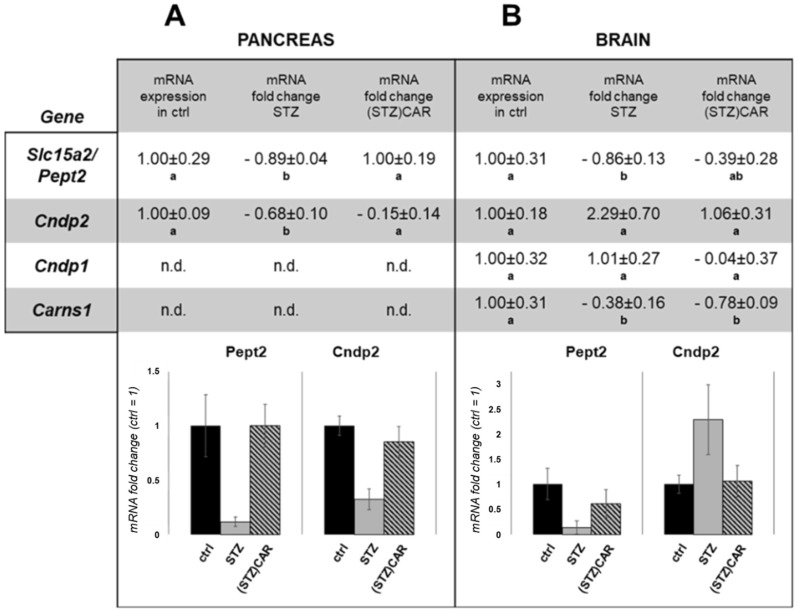
qPCR analysis of the mRNAs of genes related to CAR homeostasis (*Slc15a2*/*Pept2*, *Cndp2*, *Cndp1* and *Carns1*), in the pancreas (**A**) and brain (**B**) of hyperglycemic mice undergone STZ treatment. Ctrl: untreated control mice; STZ: hyperglycemic mice; (STZ)CAR: hyperglycemic mice orally administered with CAR. Each mRNA target expression derives from the value of the 2^−Δ*C*t^ function (*C*_t_, Threshold cycle) normalized respect to the 28S RNA [25]. n.d.: not detected mRNA expression. Histograms below emphasize the expression variations for the two mRNAs found in both tissues (i.e., Slc15a2/Pept2 and Cndp2). Values are expressed as fold change with respect to control (fold change 1). Statistical analysis: one-way ANOVA, post hoc test by Bonferroni. Different letters (a, b) indicate significantly different values (*p* < 0.05; *n* = 5).

The expression of genes related to CAR biosynthesis (i.e., *Carns1* coding for CAR synthase), degradation (i.e., *Cndp1* and *2*, CAR extra- and intracellular dipeptidases, respectively) and transmembrane transport (i.e., *Slc15a2*/*Pept2*, di-tripeptide carrier) was investigated in pancreatic tissue lysates of control (ctrl), hyperglycemic (STZ) and CAR-treated hyperglycemic mice [(STZ)CAR] by qPCR assays, the results of which are summarized in Figure 3A. Expression of the Carns1 (CAR synthesis) and Cndp1 (CAR extracellular degradation) mRNAs was not detected in all the experimental conditions. On the other hand, the mRNAs of both the *Slc15a2*/*pept2* and *Cndp2* genes were detected in control mice, and both were shown to be significantly downregulated in the pancreas of STZ mice (−0.89 ± 0.04 and −0.68 ± 0.10 negative fold change, respectively) with respect to the control (fold change 1). Both the Slc15a2/Pept2 and Cndp2 mRNA levels in the pancreas of (STZ)CAR mice were found to be not significantly different from the untreated controls. In parallel, the same mRNA expression analysis was performed in mouse brains. As reported in Figure 3B, the Carns1 mRNA levels were downregulated in both STZ and (STZ)CAR mice (−0.38 ± 0.16 and −0.78 ± 0.09 negative fold change, respectively) compared to the control. Conversely, the Cndp1 mRNA levels were not significantly affected in the different conditions. As in the pancreas, the Slc15a2/Pept2 mRNA was shown to be significantly downregulated in STZ vs. control brains (−0.86 ± 0.13 negative fold change vs. 1), while it was not significantly different in the brains of (STZ)CAR mice. Finally, the Cndp2 mRNA was slightly increased (+2.3 ± 0.70 fold change) in the brains of STZ mice, and showed no changes in the brains of (STZ)CAR mice, compared to the controls.

### 2.4. Detection of CAR Content in Pancreas and Brain Extracts

**Figure 4:**
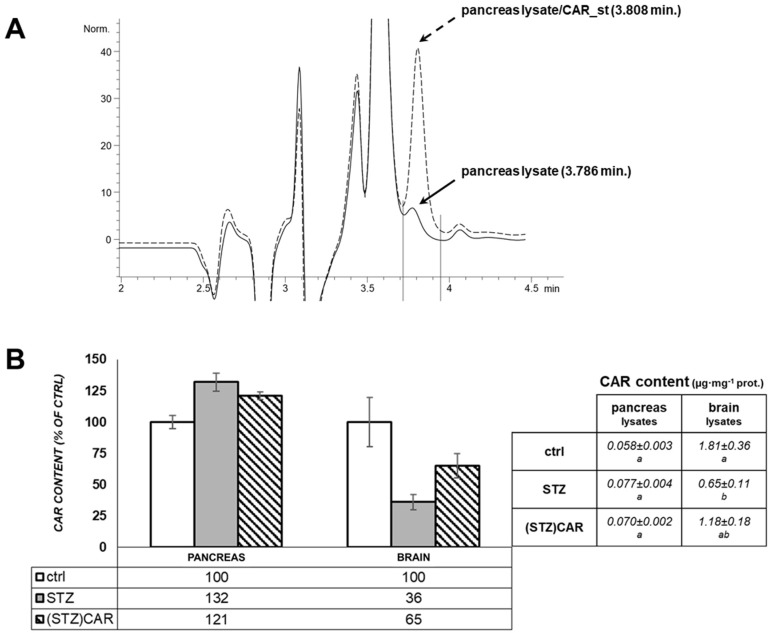
CAR detection in the pancreas and brains of mice that underwent STZ treatments. Analysis of CAR content by HPLC on deproteinized extracts of pancreas and brain lysates from control (ctrl), STZ and (STZ)CAR mice. (**A**) Representative chromatograms for the lysates from control pancreas (upper figure) and for the pancreas lysate 1:1 mixed with a CAR standard solution (1 µg/mL) in ultrapure water. Peaks and elution times are pointed by the red arrows. (**B**) Normalized concentrations of CAR per mg of total protein in the starting pancreatic extracts are expressed in the histograms as percent values with respect to the control (100%). Values are the mean of *n* = 3 (±S.E.M.) biological replicates. Different letters (a, b) indicate statistically different values (*t*-student test; *p* < 0.05).

Following gene expression analysis, the presence of CAR content was investigated in pancreatic parenchyma of mice by HPLC detection in extracts from the tissue lysates. As represented in Figure 4A, specific peaks were identified in the control pancreas (around 3.8 min elution time), which were consequently related to the presence of the CAR analyte by spiking the same samples mixed to a CAR standard solution. Moreover, the inferred quantification of the mean peak areas obtained from control, STZ, and (STZ)CAR pancreatic extracts revealed no statistically different content of CAR in the different samples; nevertheless, a slight increase in STZ-affected pancreas and a lower increase in (STZ)CAR were observed vs. controls (i.e., 132% and 121% vs. 100%). The same HPLC-based analysis was conducted on brain lysates. With respect to CAR content in untreated control brains (100%), the data indicated a decrease of up to 32% in STZ mice, and 65% in (STZ)CAR.

## 3. Discussion

In the research on CAR’s physiological roles and functions, a noticeable amount of data and studies indicates actual and/or potentially protective action exerted by CAR to prevent/ameliorate many tissue-specific diabetic complications. In rodent models, as in humans, levels of CAR have been studied in some tissues and organs in the absence or presence of diabetes onset. Moreover, levels of CAR-degrading enzymes, i.e., carnosinases, have been investigated in blood components and body fluids, and genetic links have been outlined between genes responsible for CAR degradation and diabetes [3]. Nevertheless, to date information about the expression of products of those genes involved in CAR homeostasis is still deficient in the pancreas as well as in other diabetes-targeted organs, and the identification of CAR-related gene expression alterations arising from diabetic disease states is undefined as of yet. In this framework, we aimed to shed a preliminary light to identify and characterize the networking expression of the basic genes involved in CAR in the pancreas. We adopted the diabetes model of STZ treatment in mice, comparing it with untreated mice and with STZ mice undergoing CAR dietary supplementation by mere administration in drinking water. We performed a necessary validation of our hyperglycemic model and CAR administration by monitoring (for two weeks) two systemic parameters i.e., the increase of blood glucose levels and weight loss. In both cases, STZ effects were significant and CAR administration remarkably counteracted the detrimental effects; in fact, the increase in blood glucose and weight loss were not significant when STZ-treated mice underwent CAR dietary supplementation. This evidence was further validated when we analyzed the expression of the insulin gene products in the whole pancreas of mice. We found a strong decrease of both insulin-related mRNA and protein product in the pancreatic tissue of STZ mice, which did not occur in CAR-treated STZ mice. Overall, these data agree with the literature reporting (a) the protection of the insulin-producing β cells, and (b) the hypoglycemic induction elicited by CAR [3,7]. Notably, ours are the first preliminary data on the positive modulation by CAR of the insulin gene products intrinsic in the pancreas parenchyma of mice, whereas previous works mainly report the effects of CAR on plasma insulin or insulin secretion from β-cells in culture [3,7]. Thus, in the same whole-pancreas samples we analyzed the expression of four basic CAR-related genes, by real time PCR. We identified the absence of the CAR synthesis and of the extracellular CAR dipeptidase mRNAs (Carns1 and Cndp1). Contrarily, the presence of the intracellular CAR dipeptidase (Cndp2) and the oligopeptide transmembrane transporter (Slc15a2/Pept2) mRNAs was detected. Remarkably, we could associate with the STZ impairment a significant downregulation of these gene expressions in the pancreas and, chiefly, a counteractive effect by CAR administration. Intriguingly, the expression of the two (CAR homeostasis-related) Cndp2 and Pept2 mRNAs was found in association with our first identification by HPLC of CAR occurrence in the whole pancreatic lysates; it can also be noticed that downregulation of Cndp2 (intracellular CAR degradation) mRNA induced by STZ may be related to the slight increase of CAR content, as detected by HPLC (although this is not statistically significant and thus deserves deeper investigation). Since CAR metabolism is intertwined with the homeostasis of nervous cells providing neuroprotective effects in pathophysiological situations including diabetes [3,7,8,12], we adopted the same experimental approach in a diabetes-targeted district such as CNS. In the brains of STZ-treated mice, we detected the mRNA downregulation of the CAR synthesis and transport genes (*Carns1* and *Slc15a2*/*Pept2*) together with an upregulatory trend of the intracellular CAR dipeptidase (*Cndp2*). Taken together, these mRNA variations suggest the occurrence of a dysregulation that implies the lowering of CAR levels in STZ-treated brains; intriguingly, we confirmed this via HPLC data, showing a reduced CAR content in STZ-treated brains. Remarkably, the administration of exogenous CAR to the STZ mice has counteracting (preserving) effects, i.e., it seems to partially dampen the STZ-induced effects. In fact, the CAR content in (STZ)CAR brains is enriched compared to STZ brains (even though it is still partially reduced with respect to untreated controls), as detected by HPLC. In parallel, exogenous CAR also counteracts the STZ-induced dysregulation of the Pept2 and Cndp2 mRNA expression (while it is not a stimulus to preserve the expression levels of the CAR synthesis gene *Carns1*, predictably); summing up the data in the (STZ)CAR brains, it can be hypothesized that CAR levels are supported (almost partially) by exogenous CAR, thus the expression of genes tends to follow the ‘physiological’ scheme of the untreated controls. Overall, our report contains novel hints on (a) CAR’s homeostasis (i.e., identification of CAR presence and of the CAR-related gene expression) and its impact in the mouse pancreatic parenchyma in the absence or presence of diabetic onsets, and (b) the modularity of CAR content, CAR availability, and CAR-related genes as a unified marker network along the axis between the pancreas and brain as diabetes-targeted organs.

## 4. Materials and Methods

### 4.1. Animal Handling, Ethical Approval and Treatments

Inbred male C57BL/6JB6 mice (Harlan, San Pietro al Natisone, UD, Italy) (12 weeks old) were used, under standard housing conditions. All the procedures with mice were conducted upon review and ethical approval by the Animal Care and Use Committee of the Department of Biological and Environmental Sciences and Technologies of the University of Salento, according to the EU guidelines for the ethical treatment of animals (Directive 2010/63/EU). The experimental protocols, procedures, and the number of individual mice required for the experiments were previously accepted and authorized according to the Decree n. 26/04-03-2014 of the Italian Ministry of Health, after review by the competent organs of the Veterinary Health Department. All procedures (housing, treatments, and sacrifice) were regularly checked and revised by the veterinary personnel. Three groups of five mice were treated under the following experimental conditions: (1) ctrl: untreated control mice; (2) STZ: mice treated with streptozotocin (STZ; α-anomer basis, *N*-(Methylnitrosocarbamoyl)-α-d-glucosamine, S0130 Sigma-Aldrich, Milano, Italy) dissolved in Dulbecco’s phosphate buffer saline (D-PBS), administered by intraperitoneal injection (200 mg/Kg body weight); (3) (STZ)CAR: STZ-treated mice undergoing two-week CAR (β-Alanyl-l-histidine, L-carnosine isomer, C9625 Sigma-Aldrich) administration in drinking water (1 g/L CAR in sterile deionized H_2_O) [26]. The blood glucose levels and body weight of mice were monitored every three days. Blood glucose concentrations were measured with a Contour XT glucometer (Bayer, Milano, Italy), sampling the blood by a painless skin puncture at the end of the tail. Consequent to STZ intraperitoneal injections in a total of 12 individuals, overt diabetic hyperglycemia was considered for 10 mice showing blood glucose levels ≥250 mg/dL for at least seven days, whereas two mice out of 12 showed STZ resistance and thus were excluded from the experimental protocol. After overt hyperglycemia, a group of five mice received oral CAR in drinking water ad libitum for two weeks. Sterilized CAR solutions were renewed every third day and the consumption was verified. At the end of the treatments, mice were euthanized by isoflurane inhalation overdose followed by cervical dislocation. Before extraction of the pancreas and brains, mice were terminally anesthetized with isoflurane and transcardial perfusion was performed with physiological saline solution (NaCl 0.9% *w*/*v*).

### 4.2. RNA/Protein Isolation from Tissues

After isolation and storage in RNALater (Ambion Europe, Hungtingdon, UK), pancreas and brains tissues from mice were processed for RNA and protein extraction using the AllPrep DNA/RNA/Protein mini kit (Qiagen, Milano, Italy) protocol and reagents, according to the manufacturer’s instructions. Total brain tissue lysis was performed with the AllPrep lysis buffer by using a KINEMATICA Polytron homogenizer (Thermo Fisher Scientific, Rodano, MI, Italy). RNA concentrations were calculated by spectrophotometry, and the λ260/λ280 ratios were evaluated. All the RNA extractions were qualitatively and quantitatively tested by loading RNA samples onto agarose gels. Protein extract concentrations were calculated by the Bicinchoninic Acid Kit for Protein Determination (BCA1 kit; Sigma-Aldrich), according to the manufacturer’s protocol.

### 4.3. Primer Design and Real-Time PCR Assays

For each gene, the mouse mRNA reference sequences were collected from the on-line GenBank^®^ database (at http://www.ncbi.nlm.nih.gov/) and aligned by the Clustal Omega web-based tool (http://www.ebi.ac.uk/Tools/msa/clustalo/). Exon–intron gene structure, sequence conservation, identity regions and species-specific nucleotide tracts were studied to select primer pairs for the expression analysis by qPCR assays avoiding possible genomic amplicons. Oligonucleotides were purchased from Invitrogen (Life Technologies, Monza, MB, Italy). Amplification products were sequenced and identified by alignment with the respective reference sequences. Details of the gene-specific primers used for qPCR assays are reported in Table 1. For each extracted RNA, reverse transcriptions were performed on 0.25–1 µg RNA using the Bio-Rad iScript Select cDNA Synthesis kit (Bio-Rad, Segrate, MI, Italy) according to the manufacturer’s instructions and in the presence of random primers. Before qPCR analysis, each gene-specific primer pair was tested for efficiency according to the amplification efficiency parameters for genes of interest and internal controls proposed by Schmittgen and Livak [25]. qPCR was performed using the iQ SYBR Green Supermix protocol (Bio-Rad) with a Rotor-Gene 3000 (Corbett Research, St. Neots, UK) real-time machine. To assess quantitative gene expression, 28S was used as an internal control to normalize mRNA amplifications. Gene expression relative quantification was assessed by analyzing the output threshold values (*C*_t_) according to the comparative *C*_t_ method [25]; qPCR data were shown as 2**^−^**^Δ*C*t^ values, proportional to the amount of the detected target mRNA. Δ*C*_t_ values (Δ*C*_t_ = target gene *C*_t_ − standard gene *C*_t_) were obtained from three different rounds of qPCR for both the target mRNA and the 28S internal control, of three biological replicates. Statistical analysis was performed after the 2**^−^**^Δ*C*t^ transformation [25].

### 4.4. Western Blotting

Following electrophoresis on polyacrylamide gels, proteins were electro-transferred to nitrocellulose membrane (Trans-Blot Turbo Mini Nitrocellulose Transfer packs; Bio-Rad) using a Trans-Blot Turbo Transfer System (Bio-Rad). After protein transfer, the membranes were blocked for 1 h in 5% (*w*/*v*) milk in PBS at room temperature, and then immunoblotted in 5% milk and 0.1% Tween-20 in PBS with anti-insulin antibody produced in rabbit (SantaCruz, Dallas, TX, USA). Then, blots were incubated with HRP-conjugated goat anti-rabbit secondary antibody (Sigma-Aldrich), and immune reactive bands were detected using an enhanced chemiluminescence method (ECL kit; BioRad). Densitometry was analyzed by using the NIH Image (v1.63) software (National Institutes of Health, Bethesda, MD, USA). The pixel intensity for each region was calculated, the background was subtracted, and the protein expression results were normalized with respect to the bands of an anti-α-tubulin antibody (Abcam, Cambridge, UK) as loading control for each lane.

### 4.5. CAR Content Measurement by High-Performance Liquid Chromatography (HPLC)

For the CAR content analysis by HPLC assays, pancreas and brain lysates were treated according to an optimized procedure based on previously described protocols for CAR extraction from tissue matrices [27]. In brief, frozen lysates were rapidly thawed and further homogenized by 10 passages through syringe needles. The homogenate was centrifuged at 20,000× *g* for 30 min at 4 °C and the supernatant was filtered through Whatman no. 4 filter paper. The supernatant was subjected to heat treatment at 80 °C for 15 min, subsequently cooled in an ice bath and then centrifuged at 6000× *g* for 20 min at 25 °C to remove precipitated proteins. For final deproteinization, the supernatant was mixed with 1 volume of 0.4 M perchloric acid by vortex and the mixed samples were boiled for 10 min before centrifuging at 5000× *g* for 5 min at 4 °C. The resulting supernatant was finally filtered (0.45 µm) before being processed in the HPLC run. Deproteinized samples obtained from tissues and CAR standard solutions were suitably diluted in ultrapure water (nanopure grade). The HPLC system utilized was a Hewlett-Packard module 1100 series equipped for fluorescence detection and automated sample injection. HPLC conditions were the following: (a) column Agilent Hypersyl ODS 4.6 × 250 mm (5 µm particle size); (b) flow rate 1 mL/min; (c) isocratic elution at 25 °C (mobile phase: 0.1% TFA-CH_3_CN, 98:2 *v*/*v*); (d) detection UV absorbance at 214 nm.

### 4.6. Statistical Analysis

Unless otherwise specified, differences between datasets were analyzed by one-way ANOVA and Bonferroni’s post hoc test. Differences were considered significant at *p* < 0.05. Data are reported as means ± standard error of the mean (S.E.M.).

## Figures and Tables

**Table 1:** For each gene, the NCBI accession numbers of the mRNA reference sequences (RefSeq mRNA; rDNA for the 28S) used for primer design are reported. For each (forward and reverse) primer, the 5’-3’ nucleotide sequence and the melting temperature are reported (Tm). For each mRNA detection, the expected amplicon length is indicated (PCR size) in base pairs (bp).

GENE (*Mus musclus*)	mRNA RefSeq	Forward Primer 5′–3′ (Tm)	Reverse Primer 5′–3′ (Tm)	PCR Size (bp)
*Carns1*	NM_134148	GCGGCGTCAGCAAGAAGTT(59°)	CACCAAGCAGTCATCCCAGAA(60°)	136
*Cndp2*	NM_023149.2	TTCAAGGTGTACATGGGC(54°)	AAAGGTCAAGGTCACAGGA(55°)	162
*Cndp1*	NM_177450.4	CCTAGAAGAATACCAGAAGAGC(58°)	GGGACTAGACGGATTGAAA(55°)	216
*Slc15a2*/*Pept2*	NM_021301.3	CATGAAATCTGTGCTCCAGG(57°)	AGGAGGCAGGAAAACAAAA(52°)	126
*Ins1*	NM_008386.3	TCAGAGACCATCAGCAAGCA(56°)	TCTCTACCTGGTGTGTGGG(55°)	167
*28S*	NR_003279.1	CGTGAGACAGGTTAGTTTTAC(51°)	ATCCCACAGATGGTAGCTTC(53°)	143

## References

[1] Boldyrev A.A. (1993). Does carnosine possess direct antioxidant activity?. Int. J. Biochem..

[2] Boldyrev A.A., Koldobski A., Kurella E., Maltseva V., Stvolinski S. (1993). Natural histidine-containing dipeptide carnosine as a potent hydrophilic antioxidant with membrane stabilizing function. A biomedical aspect. Mol. Chem. Neuropathol..

[3] Boldyrev A.A., Aldini G., Derave W. (2013). Physiology and pathophysiology of carnosine. Physiol. Rev..

[4] Nagai K., Niijima A., Yamano T., Otani H., Okumra N., Tsuruoka N., Nakai M., Kiso Y. (2003). Possible role of l-carnosine in the regulation of blood glucose through controlling autonomic nerves. Exp. Biol. Med..

[5] Kubomura D., Matahira Y., Nagai K., Niijima A. (2010). Effect of anserine ingestion on hyperglycemia and the autonomic nerves in rats and humans. Nutr. Neurosci..

[6] Sauerhofer S., Yuan G., Braun G.S., Deinzer M., Neumaier M., Gretz N., Floege J., Kriz W., van der Woude F., Moeller M.J. (2007). l-carnosine, a substrate of carnosinase-1, influences glucose metabolism. Diabetes.

[7] Miceli V., Pampalone M., Frazziano G., Grasso G., Rizzarelli E., Ricordi C., Casu A., Iannolo G., Conaldi P.G. (2018). Carnosine protects pancreatic beta cells and islets against oxidative stress damage. Mol. Cell. Endocrinol..

[8] Lee Y.T., Hsu C.C., Lin M.H., Liu K.S., Yin M.C. (2005). Histidine and carnosine delay diabetic deterioration in mice and protect human low density lipoprotein against oxidation and glycation. Eur. J. Pharmacol..

[9] Soliman K., Mohamed A., Metwally N. (2007). Attenuation of some metabolic deteriorations induced by diabetes mellitus using carnosine. J. Appl. Sci..

[10] Elbarbary N.S., Ismail E.A.R., El-Naggar A.R., Hamouda M.H., El-Hamamsy M. (2018). The effect of 12 weeks carnosine supplementation on renal functional integrity and oxidative stress in pediatric patients with diabetic nephropathy: A randomized placebo-controlled trial. Pediatr. Diabetes.

[11] Houjeghani S., Kheirouri S., Faraji E., Jafarabadi M.A. (2018). l-Carnosine supplementation attenuated fasting glucose, triglycerides, advanced glycation end products, and tumor necrosis factor-alpha levels in patients with type 2 diabetes: A double-blind placebo-controlled randomized clinical trial. Nutr. Res..

[12] Hipkiss A.R. (2009). Carnosine, diabetes and Alzheimer’s disease. Expert Rev. Neurother..

[13] Pavlin M., Rossetti G., De Vivo M., Carloni P. (2016). Carnosine and Homocarnosine Degradation Mechanisms by the Human Carnosinase Enzyme CN1: Insights from Multiscale Simulations. Biochemistry.

[14] Hipkiss A.R., Baye E., de Courten B. (2016). Carnosine and the processes of ageing. Maturitas.

[15] Riedl E., Pfister F., Braunagel M., Brinkkötter P., Sternik P., Deinzer M., Bakker S.J., Henning R.H., van den Born J., Krämer B.K. (2011). Carnosine Prevents Apoptosis of Glomerular Cells and Podocyte Loss in STZ Diabetic Rats. Cell Physiol. Biochem..

[16] Albrecht T., Schilperoort M., Zhang S., Braun J.D., Qiu J., Rodriguez A., Pastene D.O., Krämer B.K., Köppel H., Baelde H. (2017). Carnosine Attenuates the Development of both Type 2 Diabetes and Diabetic Nephropathy in BTBR ob/ob Mice. Sci. Rep..

[17] Janssen B., Hohenadel D., Brinkkoetter P., Peters V., Rind N., Fischer C., Rychlik I., Cerna M., Romzova M., de Heer E. (2005). Carnosine as a protective factor in diabetic nephropathy: Association with a leucine repeat of the carnosinase gene CNDP1. Diabetes.

[18] Yadav A.K., Sinha N., Kumar V., Bhansali A., Dutta P., Jha V. (2016). Association of CTG repeat polymorphism in carnosine dipeptidase 1 (CNDP1) gene with diabetic nephropathy in north Indians. Indian J. Med. Res..

[19] Peters V., Lanthaler B., Amberger A., Fleming T., Forsberg E., Hecker M., Wagner A.H., Yue W.W., Hoffmann G.F., Nawroth P. (2015). Carnosine metabolism in diabetes is altered by reactive metabolites. Amino Acids.

[20] Vardarli I., Baier L.J., Hanson R.L., Akkoyun I., Fischer C., Rohmeiss P., Basci A., Bartram C.R., van der Woude F.J., Janssen B. (2002). Gene for susceptibility to diabetic nephropathy in type 2 diabetes maps to 18q22.3–23. Kidney Int..

[21] Bowden D.W., Colicigno C.J., Langefeld C.D., Sale M.M., Williams A., Anderson P.J., Rich S.S., Freedman B.I. (2004). A genome scan for diabetic nephropathy in African Americans. Kidney Int..

[22] Bertinaria M., Rolando B., Giorgis M., Montanaro G., Guglielmo S., Buonsanti M.F., Carabelli V., Gavello D., Daniele P.G., Fruttero R. (2011). Synthesis, physicochemical characterization, and biological activities of new carnosine derivatives stable in human serum as potential neuroprotective agents. J. Med. Chem..

[23] Xie R.X., Li D.W., Liu X.C., Yang M.F., Fang J., Sun B.L., Zhang Z.Y., Yang X.Y. (2017). Carnosine Attenuates Brain Oxidative Stress and Apoptosis After Intracerebral Hemorrhage in Rats. Neurochem. Res..

[24] Wang A.H., Ma Q., Wang X., Xu G.H. (2018). Protective effects of beef decoction rich in carnosine on cerebral ischemia injury by permanent middle cerebral artery occlusion in rats. Exp. Ther. Med..

[25] Schmittgen T.D., Livak K.J. (2008). Analyzing real-time PCR data by the comparative *C*(T) method. Nat. Protoc..

[26] Herculano B., Tamura M., Ohba A., Shimatani M., Kutsuna N., Hisatsune T. (2013). β-alanyl-l-histidine rescues cognitive deficits caused by feeding a high fat diet in a transgenic mouse model of Alzheimer’s disease. J. Alzheimers Dis..

[27] Manhiani P.S., Northcutt J.K., Han I., Bridges W.C., Scott T.R., Dawson P.L. (2011). Effect of stress on carnosine levels in brain, breast, and thigh of broilers. Poult. Sci..

